# HIV epidemic and cascade of care in 12 east African rural fishing communities: results from a population-based survey in Uganda

**DOI:** 10.1186/s12889-020-09121-6

**Published:** 2020-06-19

**Authors:** J. Burgos-Soto, J. Ben Farhat, I. Alley, P. Ojuka, E. Mulogo, T. Kise-Sete, M. Bouhenia, L. Salumu, R. Mathela, C. Langendorf, S. Cohuet, H. Huerga

**Affiliations:** 1 Epicentre, 14-34 Avenue Jean Jaures, 75019 Paris, France; 2 Epicentre, Mbarara, Uganda; 3 Mbarara University of Science and Technology, Mbarara, Uganda; 4 Ministry of Health of Uganda, Rubirizi, Uganda; 5 Médecins sans Frontières, Paris, France; 6 Medecins sans Frontières, Kampala, Uganda

**Keywords:** HIV, Cascade of care, Epidemic, Fishing, Communities, Africa, Antiretroviral therapy, Diagnosis, Viral suppression

## Abstract

**Background:**

In East Africa, fishing communities are considered most-at-risk populations for the acquisition of HIV. We estimated HIV prevalence and assessed progress towards the UNAIDS 90–90-90 targets along the HIV treatment cascade in 12 fishing communities surrounding Lakes Edward and George, Uganda.

**Methods:**

We conducted a cross-sectional household-based survey between September and November 2016. All adults between 15 and 69 years old were eligible to participate. Children below 15 years old were eligible for HIV testing if either parent was HIV-positive. Viral load testing was done for all HIV-infected individuals. Logistic regression models adjusted for sociodemographic-behavioral variables were used to assess the association between occupation and HIV positivity.

**Results:**

Overall, 1738 adults (959 women, 779 men) and 148 children were included. Adult inclusion rate was 96.0%. Of the men, 58% reported to be fishermen. The HIV-prevalence among adults was 17.5% (95%CI: 15.8–19.4) and 6.1% (95%CI: 3.1–11.4) among HIV-exposed children. HIV prevalence was higher among women than among men (20.9% vs. 13.5%, *p* < 0.001). Among men, fishermen had a higher HIV prevalence (18.7%; 95%CI: 15.1–22.3) and a higher risk of being HIV-positive (aOR: 4.2; 95%CI: 2.0–9.1) than men of other occupations (*p* < 0.001). Progress towards the UNAIDS 90–90-90 targets was as follows: 86.5% (95%CI: 82.3–90.1%) of the HIV-positive participants were diagnosed, 98.7% (95%CI: 96.1–99.6%) of those aware were on antiretroviral therapy (ART), and 87.3% (95%CI: 82.3–91.0%) of those on ART were virally suppressed. Overall, 73% of all HIV-positive individuals were virally suppressed. Viral suppression was lower among individuals 15–24 years (45.5%) than among those 25–44 years (74.0%) and 45–69 years (85.0%), *p* < 0.001. Fishermen did not to have significant differences in the HIV cascade of care compared to men with other occupations.

**Conclusions:**

HIV prevalence was high in these fishing communities, particularly among women and fishermen. Important progress has been made along the HIV treatment cascade, and the UNAIDS goal for viral suppression in population was achieved. However, gaps remain and HIV care strategies focusing on young people are urgently needed. HIV preventive interventions should target particularly women, young people and fishermen though HIV preventive and care services should remain available to the whole fishing communities.

## Background

In 2019, sub-Saharan Africa is still the region with the highest burden of HIV worldwide, hosting 21 million people living with HIV/AIDS [1]. Despite this high burden, important progress in rolling back the HIV epidemic has been made during the last years, particularly in Eastern and Southern Africa. By 2019 the number of new HIV-infections and AIDS-related deaths has declined by 28.0 and 44.0% respectively in these regions [1, 2]. Although the epidemic picture has improved in these regions, only the first indicator of the HIV cascade of care (testing) is above 80%. The other two (being on antiretroviral therapy and viral suppression) are still below 70% in eastern and southern Africa [1, 2].

Most of the new HIV infections in sub-Saharan Africa occur in the general population [3], however, most-at-risk populations (MARPs) continue to contribute importantly to the epidemic burden in this region. MARPs are groups that engage in behaviors and practices that put them at a higher risk of HIV exposure and, in Eastern-Africa, fisher folks are considered part of these groups [4].

According to current estimates, HIV-prevalence in fishing communities surrounding Lake Victoria is high, ranging between 22.0 and 29.0% [5–9]. The high burden of HIV in these fishing communities is owed to context-specific sociodemographic and structural factors. Fishing is mostly conducted by men whereas women carry out the commercialization of the catch. This labor organization favors gender imbalances, placing women at a higher risk of contracting HIV infection through transactional sex in exchange for merchandise [7, 9–11]. Moreover, as fishing is a seasonal activity, fishermen and markets move periodically between fishing spots, and this nomadic behavior presents a major challenge to HIV program enrollment and retention [9, 11].

Uganda’s HIV care program includes HIV Counselling and Testing campaigns done via outreach clinics in the fishing communities surrounding Lakes Victoria, George and Edward. However, challenges to delivering treatment remain. Thus, in order to strengthen Uganda’s HIV care program, particularly in the fishing communities surrounding Lakes George and Edward, *Médecins Sans Frontières* (MSF) runs a public health intervention to increase access to HIV counseling and testing services and improve clinical care of HIV-infected individuals living in these settings. This public health intervention, launched in 2016, had two main activities: first, an HIV screening campaign at the landing sites of fishing communities and, second, providing technical support to local health centers offering HIV care and treatment to these communities.

As we start the deadline year for the Joint United Nations Programme on HIV/AIDS (UNAIDS) ambitious Global Plan to achieve 90% of HIV-infected people aware of their serological status, 90% of HIV-infected people receiving antiretroviral therapy (ART) and 90% virally suppressed by 2020, knowledge of HIV care cascade among MARPs remains scarce. Although fishing is a common activity in East Africa there is a paucity of robust data on the HIV epidemic in fishing communities, particularly in setting others than Lake Victoria. While some data on HIV prevalence in these populations is available, it rarely includes the HIV treatment cascade indicators. To design an effective, context-adapted public health intervention for these communities, MSF needed a more detailed understanding of the HIV epidemic. We conducted an HIV population-based survey in the fishing communities surrounding Lakes George and Edward in Uganda in order to estimate the prevalence of HIV and indicators along the HIV care cascade. Secondly, we aimed to assess individual characteristics associated with being HIV untested and virally unsuppressed in these communities.

## Methods

### Design and population

Between September and November 2016, we conducted a cross-sectional household-based survey in 12 fishing communities surrounding Lakes Georges and Edward in Uganda, where MSF implemented a community-based public health intervention. All women and men aged 15–69 years old who were living in these communities and visitors who slept in the household the night before the survey were eligible to participate. Children under 15 years old were eligible if they were HIV-exposed (one of the parents was HIV positive).

### Sample size and sampling procedures

A total of 890 households from the 12 landing sites participating in the survey were randomly selected to achieve a sample size of at least 1537 adults (expected average of 1.8 adults per household included in the study and 5% of the selected households not included). This sample size was calculated to have enough power to estimate an HIV prevalence of 10% with a precision of 1.5%. No sample size was calculated for children. All children fulfilling the study eligibility criteria and living in the households selected for the study were proposed to participate in the study. As a sampling frame for the random selection of households, we conducted an exhaustive household enumeration in the 12 landing sites. Households were enumerated using Global Positioning System (GPS) devices (GARMIN Etrex). The spatial coordinates of each household enumerated were coded and recorded in the GPS devices and verified and stored in an electronic database. Simple random sampling to identify households for inclusion was done using the geosampling method.

### Data collection process

Local nurses were recruited and trained on the methodological, logistic and clinical aspects of the survey, including HIV counseling and testing. For the field work, nurses were organized into four teams and assigned each to one landing site at time. Each team was provided with visits charts listing the coded spatial coordinates of the randomly selected households and GPS devices with the coordinates of households per landing sites.

Once in the household, the nurse identified the household head, presented the survey and requested written consent for participation in the study. The head of household was then asked to list all the residents of the household as well as the visitors who slept in the household the night prior to the survey. The interviewer verified the study eligibility criteria for each household member.

Eligible adults were invited to provide written informed consent for study participation and continue with individual interviews. Individual questionnaires, specifically developed for this study, collected information about the sociodemographic and health background of each respondent (Additional file 1). After the interview, a trained nurse performed an HIV test according to Ugandan HIV counseling and testing protocols (sequential use of up to 3 different HIV tests: Abbott Determine HIV 1/2, Stat Pack HIV Chembio and Uni-Gold™ Recombigem HIV Trinity Biotech). HIV-positive individuals were asked about their HIV status awareness, HIV care and antiretroviral treatment (including date and place of the first HIV test, the last HIV consultation, the start of ART and the stop of ART for those who had stopped taking treatment). HIV positive individuals were also asked to provide a sample of 4 ml of whole blood for further viral load testing. The blood samples were collected in a vacutainer EDTA tube and sent to a reference laboratory in Mbarara for viral load quantification (Roche Taqman 48).

Eligible children whose parents provided written informed consent for study participation were also tested for HIV. Children over 18 months, followed the same protocol than adults for HIV testing. Children 18 months and below were tested using a DNA Polymerase Chain Reaction (PCR), as per Uganda’s HIV counseling and testing guidelines. Therefore, for this population a venous blood sample of 1 ml was collected, sent to the reference laboratory and tested using test Roche Taqman 48.

### Data management and statistical analysis

Data collected on paper forms was entered into an electronic database (Epidata version 3.1) and analyzed using Stata 13 (Stata Corp., College Station, Texas, USA). To describe the study population, variables were presented as proportions. Differences between groups were tested using Chi-squared test. We estimated HIV prevalence with 95% confidence intervals (CI). HIV cascade of care indicators were calculated and presented using the UNAIDS 90–90-90 targets definitions. HIV diagnosis rate (1st 90) was defined as the proportion of HIV-positive individuals who reported that they were aware of their HIV status prior to being tested during the survey. ART rate (2nd 90) was defined as the proportion of individuals on ART among those aware of their HIV-positive status. Viral suppression rate (3rd 90) was defined as the proportion of individuals with viral load less than 1000 copies/mL among those on ART.

We used logistic regression models to identify individual factors associated with being HIV-positive among women and men and, being untested for HIV in the previous 12 months among HIV-negative participants. Variables for adjustment included in the analyses were: age, sex, marital status, education level, occupation, time living in the landing site, place of residence, time slept away from home. In addition, in the models for being HIV-positive, condom use and male circumcision were also included as variables for adjustment. Univariate logistic regression analyses were conducted to assess the association with the dependent variables. Variables associated with dependent outcomes at *p*-values of 0.20 were retained for multivariate regression models. A backward stepwise method was used in the multivariate regression analyses to identify a final set of explanatory variables. *P*-values under 0.05 were considered statistically significant for final multivariate logistic models. Adjusted Odds Ratios (aOR) were presented.

### Ethical aspects and agreements

The protocol, informed consent forms and questionnaires in English and Lukonzo, were approved by the Mbarara University of Science and Technology (MUST) Ethics Committee, and the Uganda National Council for Science and Technology (UNCST). Written informed consent to participate in the survey was obtained from all adult participants and from mature minors (aged ≥15 years). Written informed consent was also obtained from a parent or guardian on behalf of participants aged below 15 years. All written consents were provided to participants in English or in Lukonzo (local language at the study setting) according to participant preferences.

## Results

### Eligibility & inclusions

**Fig. 1:**
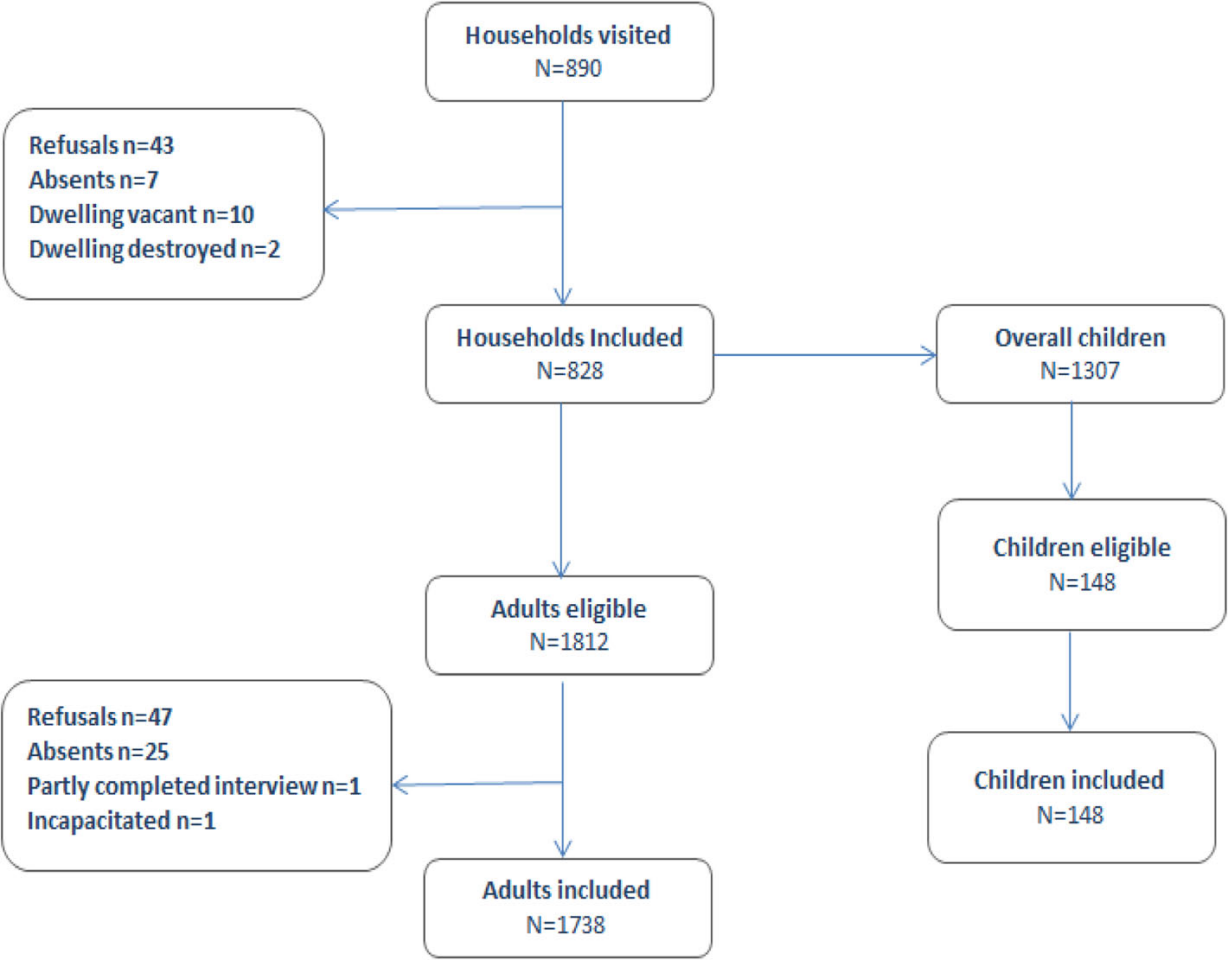
Flow chart study population

Overall, a total of 7436 households were identified and enumerated across the 12 landing sites. Of the 890 randomly selected households, 828 were included (household inclusion rate: 93.0%). Refusal and absence rate at the household level were of 4.8 and 0.7% respectively. On average, there were 2.1 adults per household eligible to participate in the survey. Of the 1307 children identified in the households included in the study, 148 (11.3%) were eligible to participate in the survey (Fig. 1).

At the individual level, 1812 adults (980 women and 832 men) were eligible to participate in the survey, and 1738 (959 women and 779 men) were included. The overall individual adult inclusion rate was 96.0% and the inclusion rates among women and men were 97.9 and 93.6% respectively. The 148 children eligible to participate in the survey were included (individual children inclusion rate: 100%).

### Study population

Of the 1738 individuals included in the study, 959 were women (55.2%). Median age was 30 years (IQR: 22–40). Overall, 35.2% of the adults reported being residents of the landing site for less than 6 years and 2.8% were visitors. Half of adults interviewed (50.1%) reported sometimes sleeping away from home. Among them, the proportion of men sleeping away from home for 3 months or more was significantly higher than that of women (men: 32.1% vs. women: 17.8%, *p* < 0.001). The most common occupation among men was fishing (58.4%) and among women was sales and service work (35.4%) (Table 1).

**Table 1:** Sociodemographic and behavior profile of the study population by sex

	Total (***N*** = 1738)		Women (***N*** = 959)		Men (***N*** = 779)		***P***-value
	n	%	n	%	n	%	
**Age**							
[15–24 years old]	587	33.8	368	38.4	219	28.1	< 0.001
[25–44 years old]	849	48.9	458	47.8	391	50.2	
[45–69 years old]	302	17.4	133	13.9	169	21.7	
**Education level**							
Never attended school	264	15.2	189	19.7	75	9.6	< 0.001
Primary level or higher	1474	84.8	770	80.3	704	90.4	
**Marital status**							
Single	323	18.6	156	16.3	167	21.4	< 0.001
Married/In union	1121	64.5	587	61.2	534	68.5	
Divorced/Separated/ Widowed	294	16.9	216	22.5	78	10.0	
**Place of current residence**							
Kasese	818	69.4	462	69.1	356	70.0	0.19
Rubirizi	135	11.5	68	10.2	67	13.2	
Kamwenge	191	16.2	117	17.5	74	14.5	
Other	34	2.9	22	3.3	12	2.4	
**Time living in the landing site**							
Always	560	32.2	290	30.2	270	34.7	0.001
< = 6 years	611	35.2	372	38.8	239	30.7	
> 6 years	517	29.7	264	27.5	253	32.5	
Visitor	50	2.9	33	3.4	17	2.2	
**Time slept away from home**							
Never slept away	867	49.9	515	53.7	352	45.2	< 0.001
< =3 Months	655	37.7	365	38.1	290	37.2	
> 3 Months	216	12.4	79	8.2	137	17.6	
**Occupation**							
Fishing	471	27.1	16	1.7	455	58.4	0.001
Sales and service worker	465	26.8	339	35.3	126	16.2	
Farmer	214	12.3	158	16.5	56	7.2	
Housewive	240	13.8	240	25.0	–	–	
Student	113	6.5	50	5.2	63	8.1	
None	95	5.5	80	8.3	15	1.9	
Salt miners	88	5.1	55	5.7	33	4.2	
Other^a^	52	3.0	21	2.2	31	4.0	
**HIV test in lifetime**							
Ever tested	1618	93.4	915	95.7	703	90.2	0.01
Never tested	114	6.5	41	4.3	73	9.4	
**Condom use**							
Always	88	5.1	44	4.6	44	5.6	0.32
Never or sometimes	1650	94.9	915	95.4	735	94.4	
**Male circumcision**							
Yes	–	–	–	–	627	80.5	
No	–	–	–	–	152	19.5	

^a^Other includes: Soldier, police, factory worker, clerical, professional manager

### HIV preventive behaviors and HIV testing

Overall, 95% of the adults reported inconsistent condom use (never or sometimes). The majority of men (80.5%) were circumcised, 40.5% of them before the age of 5 years old. Half of the men (49.3%) were aware of the HIV preventive benefits of circumcision.

Overall, 93.4% of adults reported having an HIV test at least once in their lifetime and among HIV-negative adults, 81.0% reported having an HIV test within the last 12 months. In the multivariable analyses, after adjustment, HIV-negative men had a higher risk of being untested within the 12 months prior to the survey compared to women (aOR: 2.0, 95%CI: 1.4–2.8, *p* < 0.001). The majority of adults (69.5%) had their last HIV test in a public health facility and 12.9% reported that they had their last HIV test through MSF services.

### HIV prevalence

Overall, 305 individuals had reactive HIV test results, resulting in an HIV prevalence of 17.5% (95%CI: 15.8–19.4). The HIV prevalence was higher among women than among men (20.9%; 95%CI: 18.4–23.5 vs. 13.5%; 95%CI: 11.3–16.1; *p* < 0.001) (Table 2). Among children exposed to HIV, the prevalence was 6.1% (95%CI: 3.1–11.4). In terms of occupation, among women, there was no statistical difference in the HIV prevalence by occupation. However, among men, fishermen had the highest HIV prevalence (18.7%) and a 4 times higher risk of being HIV-positive in multivariable models (aOR: 4.2; 95%CI: 2.0–9.1) compared to men with other occupations. Uncircumcised men also had a higher risk of being HIV-positive (aOR: 3.5, 95%CI: 1.9–6.5, *p* < 0.001).

**Table 2:** HIV prevalence in study population groups, by sex

	Total (***N*** = 1738)		Women (***N*** = 959)		Men (***N*** = 779)		
	n	%	n	%	n	%	***P***-value
**All HIV-positive**	305	17.6	200	20.9	105	13.5	< 0.001
**Age**							
15–24 years old	33	5.6	30	8.2	3	1.4	0.001
25–44 years old	210	24.7	143	31.2	67	17.1	< 0.001
45–69 years old	62	20.5	27	20.3	35	20.7	0.93
**Marital status**							
Single	15	4.6	12	7.7	3	1.8	0.01
Married/In union	223	19.9	133	22.7	90	16.9	0.02
Divorced/Separated/ Widowed	67	22.8	55	25.5	12	15.4	0.07
**Place of current residence**							
Kasese	142	17.4	98	21.2	44	12.4	0.001
Rubirizi	38	28.2	19	27.9	19	28.4	0.96
Kamwenge	43	22.5	32	27.4	11	14.9	0.04
Other	9	26.5	7	31.8	2	16.7	0.34
**Time living in the landing site**							
Always	73	13.0	44	15.2	29	10.7	0.12
< = 6 years	113	18.5	85	22.9	28	11.7	0.001
> 6 years	112	21.7	64	24.2	48	19.0	0.15
Visitor	7	14.0	7	21.2	–		–
**Time slept away from home**							
Never slept away	139	16.0	97	18.8	42	11.9	0.007
< =3 Months	128	19.5	90	24.7	38	13.1	< 0.001
> 3 Months	38	17.6	13	16.5	25	18.3	0.74
**Occupation**							
Fishing	88	18.7	3	18.8	85	18 .7	0.99
Sales and service worker	90	19.4	82	24.2	8	6.4	< 0.001
Farmer	50	23.4	44	27.9	6	10.7	0.009
Housewive	44	18.3	44	18.3	–	–	–
Student	1	0.9	1	2.0	–	–	–
None	19	20.0	18	22.5	1	6.7	0.16
Salt miners	8	9.1	6	10.9	2	6.1	0.44
Other^a^	5	9.6	2	9.5	3	9.7	0.99
**Condom use**							
Always	28	31.8	18	40.9	10	22.7	0.07
Never or sometimes	277	16.8	182	19.9	95	12.9	< 0.001
**Male circumcision**							
Yes					56	8.9	
No					49	32.2	

^a^Other includes: Soldier, police, factory worker, clerical, professional manager

### HIV cascade of care (90–90-90 targets)

Indicators along the HIV treatment cascade were estimated as follows: 86.2% (95%CI: 81.9–89.7) of all HIV-positive individuals were aware of their status, 98.7% (95%CI: 96.1–99.6) of those diagnosed were on ART and, 87.3% (95%CI: 82.3–91.0) of those on ART were virally suppressed. Overall, 73.1% (95%CI: 67.7–77.8) of all HIV-positive individuals were virally suppressed (Table 3).

**Table 3:** HIV cascade of care indicators (90–90-90 targets and viral suppression in population) in study population groups

	Diagnosed among all HIV-positive		On ART among HIV-positive diagnosed		Virally suppressed among HIV-positive on ART		Virally suppressed among all HIV-positive	
	n/N	% (95%CI)	n/N	% (95%CI)	n/N	% (95%CI)	n/N	% (95%CI)
**All**	263/305	86.2 (81.9–89.7)	235/238	98.7 (96.1–99.6)	199/228	87.3 (82.3–91.0)	217/297	73.1 (67.7–77.8)
**Women**	173/200	86.5 (81.0–90.6)	158/159	99.4 (95.6–99.9)	137/156	87.8 (81.6–92.1)	151/198	76.3 (69.8–81.7)
**Men**	90/105	85.7 (77.6–91.2)	77/79	97.5 (90.3–99.4)	62/72	86.1 (75.9–92.4)	66/99	66.7 (56.7–75.3)
**15–24 years**	25/33	75.8 (58.1–87.6)	21/21	100.0	15/21	71.4 (48.5–86.9)	15/33	45.5 (29.3–62.6)
**25–44 years**	179/210	85.2 (79.7–89.4)	159/160	99.4 (95.6–99.9)	135/154	87.7 (81.4–92.0)	151/204	74.0 (67.5–79.6)
**45–69 years**	59/62	95.2 (85.9–98.5)	55/57	96.5 (86.8–99.1)	49/53	92.5 (81.3–97.2)	51/60	85.0 (73.4–92.1)
**Men fishermen**	76/88	86.4 (77.4–92.1)	64/66	97.0 (88.5–99.3)	53/60	88.3 (77.3–94.4)	56/83	67.5 (56.6–76.7)
**Men with other occupations**	17/20	85.0 (61.3–95.3)	16/16	100.0	12/15	80.0 (51.4–93.8)	13/19	68.4 (44.2–85.6)

**Fig. 2:**
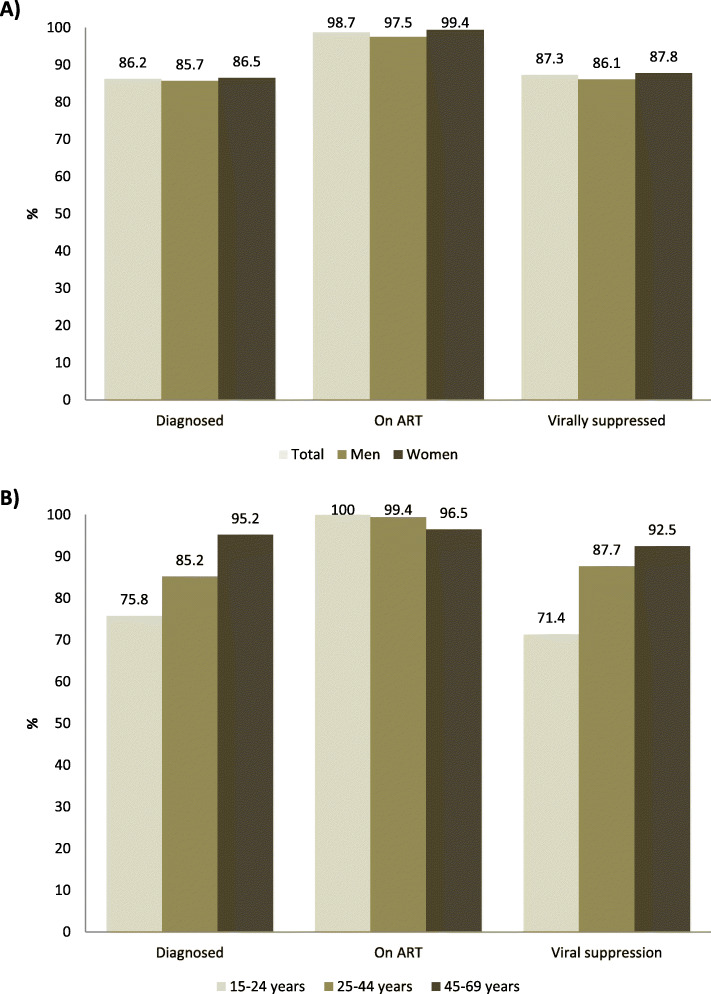
HIV cascade of care indicators (90–90-90 targets) of HIV-infected individuals of fishing communities surrounding Lake Edward and Lake George, Uganda by sex (Panel **a**) and by age group (Panel **b**). Diagnosed (1st 90) is the proportion of HIV-positive aware of their serological status among all HIV-positive. On ART (2nd 90) is the proportion of HIV-positive on ART among those aware. Virally suppressed (3rd 90) is the proportion of HIV-positive virally suppressed among those on ART

Disaggregating the HIV care cascade by sex, there were no statistically significant differences by sex (Fig. 2a). HIV diagnosis rates were 86.5 and 85.7% among women and men respectively (*p* = 0.85). ART rates among individuals diagnosed were 99.4 and 97.5% among women and men respectively (*p* = 0.22). Viral suppression rate among individuals on ART was 87.8 and 86.1% among women and men respectively (*p* = 0.72). However, viral suppression among all HIV-positive tended to be lower among men than among women: 66.7 and 76.3% respectively (*p* = 0.08).

Disaggregating the HIV care cascade by age, the proportion of individuals diagnosed was significantly lower among individuals under 25 years old compared to older adults (Fig. 2b). HIV diagnosis rate was 75.8% among individuals 15–24 years, 85.2% among those 25–44 years and 95.2% among individuals 45–69 years (*p* = 0.03). There was no statistically significant difference in the proportion of adults on ART among those diagnosed by age (*p* = 0.21). The proportion of HIV-positive individuals on ART that were virally suppressed was significantly lower among those under 25 years (71.4%) compared to their older counterparts (25–44 years: 87.7% and 45–69 years: 92.5%), *p* = 0.05. Viral suppression among all HIV-positive was lower among individuals 15–24 years (45.5%) than among those 25–44 (74.0%) and 45–69 years (85.0%), *p* < 0.001.

Disaggregating the HIV care cascade in men by occupation, there were no statistically significant differences comparing the cascade of care rates of fishermen and men with other occupations (Table 3). Viral suppression among all HIV-positive was also similar among fishermen and among men with other occupations: 67.5% versus 68.4% respectively (*p* = 0.86).

## Discussion

The present population survey answered several key questions about the HIV epidemic in the fishing communities of rural areas in East Africa. Overall the prevalence of HIV in the fishing communities surrounding Lakes George and Edward in Uganda was high (17.5%). HIV prevalence was higher in women than in men. Fishermen had a higher risk of being HIV-positive than men of other occupations. Overall, access to HIV testing services was good, as indicated by the high proportion of individuals (93.4%) reporting at least one HIV test in their lifetime. HIV-negative men presented an increased risk of being untested for HIV in the 12 months prior to the survey compared to women. Regarding the HIV care and treatment cascade, there was considerable progress towards the 90–90-90 UNAIDS goals: 86% of HIV-positive were diagnosed, 99% of those diagnosed were on ART and 87% of those on ART were virally suppressed. Overall, 73% of all HIV-positive individuals were virally suppressed, achieving UNAIDS goal for viral suppression in population. Despite this progress, several gaps remained; young people 15–24 years had lower rates than older individuals at almost all stages of the cascade of care and a very low viral suppression rate in population (45%), and men tended to have a lower viral suppression compared to women. Fishermen did not have significant differences in the HIV cascade of care compare to men with other occupations.

In terms of epidemic burden, the 17.5% prevalence of HIV in the fishing communities surrounding Lakes George and Edward in Uganda found in this survey was lower than in other fishing communities surrounding lake Victoria (28.8%) [5–9] but higher than in general population in Uganda, estimated of 6.2% [12]. According to our findings, women and fishermen appeared to have the highest epidemic burden in these communities, suggesting a particular vulnerability and a possible lack of knowledge and tools to prevent HIV.

The assessment of the HIV cascade of care in these fishing communities showed that although some indicators were slightly below the 90–90-90 UNAIDS targets, considerable progress towards these targets has been made and overall target for viral suppression in population was achieved. When compared to similar epidemiological assessment conducted during the same period, levels of HIV diagnosis and ART initiation were higher than in southern African settings [13–16]. However, viral suppression rate in fishing communities was lower than in other southern and eastern Africa countries [13–18]. Several HIV counselling and testing campaigns had been conducted in the area before the survey and may have contributed to the relatively high rate of HIV awareness. Ministry of Health (MOH) implementing partners were conducting a campaign called “know your status awareness” in Kamwenge District at the time of the survey and MSF had also started a door-to-door HIV testing campaign in Kasese District. In addition, few months prior to conducting the survey, MSF had started offering ART in the area via mobile clinics. Other strategies implemented after the survey, such as scaled up Assisted Partner Notification, “Moon Light HIV testing” in hot spots, HIV self-testing, community HIV testing services, Test and Treat, clinic days for fisher folks, accreditation of facilities on the landing sites to offer, increased availability of antiretroviral drugs, differentiated service delivery, information points to give education on HIV infection and adherence and peer led strategies, are expected to increase further the number of individuals diagnosed, treated with ART and virally suppressed.

Lower rates of HIV diagnosis, treatment and viral suppression were observed among individuals 15–24 years of age, similar to findings in other southern and eastern Africa settings [13–21]. As in other sub-Saharan Africa settings, men of the fishing communities also presented lower viral suppression rates compared to women [13–16, 18, 21]. It has been pointed out that HIV/AIDS program response for men seems to be less successful than for women, with men having higher mortality rates [22, 23]. However, women constitute the group with the highest HIV care needs due to the HIV burden in this group. Proposing HIV services adapted to the individual characteristics (age and gender) and to the living circumstances of these communities would help to increase access to HIV testing and care.

This study has some limitations. Due to the cross-sectional design of the study, we could not establish causal relationships between participants’ characteristics or behaviors and the acquisition of HIV infection. In addition, information on risk behavior such as multi-partnership or transactional sex likely associated to the spread of HIV epidemic in this setting was not captured in this study. Finally, we did not collect information on ART adherence or assess for virological failure in individuals who were virologically unsuppressed. The present population survey is amongst the first to be conducted in rural, remote fishing communities other than Lake Victoria in East-Africa. This is may be among the first studies presenting a full assessment of the HIV cascade of care in such settings. However, larger, prospective or qualitative assessments could help to fully understand the epidemic dynamics and the reasons for HIV cascade gaps found in this setting.

## Conclusions

HIV prevalence was high in the fishing communities surrounding Lakes George and Edward, and particularly among women and fishermen. Important progress has been made along the HIV treatment cascade, and the UNAIDS goal for viral suppression in population was achieved. However, there are still gaps among young people. To close these gaps, HIV care strategies focusing on young people are urgently needed. HIV preventive interventions should target particularly women, young people and fishermen though HIV preventive and care services should remain available to the whole fishing communities.

## Supplementary information

**Additional file 1.** Survey questionnaire: Household, men and women questionnaires.

## Data Availability

The datasets used and/or analysed during the current study are available from the corresponding author on reasonable request.

## Abbreviations

ART: Antiretroviral therapy
MARPs: Most-at-risk populations
MSF: Médecins Sans Frontières
UNAIDS: Joint United Nations Programme on HIV and AIDS
PCR: Polymerase Chain Reaction
CI: Confidence interval
aOR: Adjusted Odds Ratio
MUST: Mbarara University of Science and Technology
UNSCT: Uganda National Council for Science and Technology
MOH: Ministry of Health
