# Supplementary material for: HIV epidemic and cascade of care in 12 east African rural fishing communities: results from a population-based survey in Uganda
Source: BMC Public Health. 2020 Jun 19;20:970. doi: 10.1186/s12889-020-09121-6 (PMC7305611; doi:10.1186/s12889-020-09121-6)
Supplement: Supplementary file 1 — Additional file 1. Survey questionnaire: Household, men and women questionnaires. [file 12889_2020_9121_MOESM1_ESM.pdf]

Lake Edward/ Lake George HIV Seroprevalence Survey -2015 - Household questionnaire
UGANDA -KASESE DISTRICT
EPICENTRE

IDENTIFICATION (1)

LANDING SITE ____________________

VILLAGE ____________________

CONTACT/PHONE NUMBER ____________________

HOUSEHOLD NUMBER . . . . [ ][ ][ ]

INTERVIEWER VISITS

	1	2	3	FINAL VISIT
DATE	________	________	________	DAY [ ][ ]
				MONTH [ ][ ]
				YEAR [ ][ ][ ][ ]
INTERVIEWER'S NAME	________	________	________	INT. ID [ ][ ]
RESULT*	________	________	________	RESULT* [ ][ ]
NEXT VISIT: DATE	________	________		TOTAL NUMBER OF VISITS [ ]
TIME	________	________		

*RESULT CODES:

1	COMPLETED
2	NO HOUSEHOLD MEMBER AT HOME OR NO COMPETENT RESPONDENT AT HOME AT TIME OF VISIT
3	ENTIRE HOUSEHOLD ABSENT FOR EXTENDED PERIOD OF TIME
4	POSTPONED
5	REFUSED
6	DWELLING VACANT OR ADDRESS NOT A DWELLING
7	DWELLING DESTROYED
8	DWELLING NOT FOUND
98	OTHER ____________________ (SPECIFY)

TOTAL PERSONS IN HOUSEHOLD [ ][ ]

TOTAL ELIGIBLE WOMEN [ ][ ]

TOTAL ELIGIBLE MEN [ ][ ]

LINE NO. OF RESPONDENT TO HOUSEHOLD QUESTIONNAIRE [ ][ ]

SUPERVISOR	OFFICE EDITOR
NAME ________ [ ]	[ ][ ]

THIS PAGE IS INTENTIONALLY BLANK

HOUSEHOLD SCHEDULE

LINE NO.	RESIDENTS AND VISITORS	RELATIONSHIP TO HEAD OF HOUSEHOLD	SEX	RESIDENCE		AGE	DATE OF ARRIVAL	ELIGIBILITY	IDENTIFICATION
**1**	**2**	**3**	**4**	**5**	**6**	**7**	**8**	**9**	**10**
	Please give me the first names of the persons who usually live in your household and guests of the household who stayed here last night, starting with the head of the household.<br>AFTER LISTING THE NAMES AND RECORDING THE RELATIONSHIP AND SEX FOR EACH PERSON, ASK QUESTIONS 2A-2C TO BE SURE THAT THE LISTING IS COMPLETE.<br>THEN ASK APPROPRIATE QUESTIONS IN COLUMNS 5-11 FOR EACH PERSON.	What is the relationship of (NAME) to the head of the household?<br>SEE CODES BELOW.	Is (NAME) male or female?	Does (NAME) usually live here?<br>RESIDENT DEFINI	Did (NAME) stay here last night?	How old is (NAME)?<br>IF LESS THAN 1 YEAR, RECORD'00'<br>IF 95 OR MORE, RECORD '95'.	Since when did (NAME) arrive in the household?<br>1= <1 WEEK<br>2=<1 MONTH<br>3= <6 MONTHS<br>4= <1 YEAR<br>5= >1 YEAR	Is (NAME) eligible?<br>(A resident is eligible if age between 15-69)	STICK INDIVIDUAL ID NUMBER FOR EACH ELIGIBLE RESIDENT AND VISITOR AGE 15-69
			M F	Y N	Y N	IN YEARS		Y N	
01			1 2	1 2	1 2			1 2	
								Y N	
02			1 2	1 2	1 2			1 2	
								Y N	
03			1 2	1 2	1 2			1 2	
								Y N	
04			1 2	1 2	1 2			1 2	
								Y N	
05			1 2	1 2	1 2			1 2	
								Y N	
06			1 2	1 2	1 2			1 2	

07			1 2	1 2	1 2			Y N<br>1 2	
08			1 2	1 2	1 2			Y N<br>1 2	
09			1 2	1 2	1 2			Y N<br>1 2	
10			1 2	1 2	1 2			Y N<br>1 2	

CODES FOR Q. 3: RELATIONSHIP TO HEAD OF HOUSEHOLD

01 = HEAD
02 = WIFE OR HUSBAND
03 = SON OR DAUGHTER
04 = SON-IN-LAW OR DAUGHTER-IN-LAW
05 = GRANDCHILD
06 = PARENT
07 = PARENT-IN-LAW
08 = BROTHER OR SISTER
09 = OTHER RELATIVE
10 = ADOPTED/FOSTER/
11 = NOT RELATED
99 = DON'T KNOW

LINE NO.	RESIDENTS AND VISITORS	RELATIONSHIP TO HEAD OF HOUSEHOLD	SEX	RESIDENCE		AGE	DATE OF ARRIVAL	ELIGIBILITY	IDENTIFICATION
1	2	3	4	5	6	7	8	9	11
			M F	Y N	Y N	IN YEARS		Y N	
11			1 2	1 2	1 2			1 2	
								Y N	
12			1 2	1 2	1 2			1 2	
								Y N	
13			1 2	1 2	1 2			1 2	
								Y N	
14			1 2	1 2	1 2			1 2	
								Y N	
15			1 2	1 2	1 2			1 2	
								Y N	
16			1 2	1 2	1 2			1 2	
								Y N	
17			1 2	1 2	1 2			1 2	
								Y N	
18			1 2	1 2	1 2			1 2	

19		☐☐	1 2	1 2	1 2	☐☐	☐	Y N<br>1 2	
20		☐☐	1 2	1 2	1 2	☐☐	☐	Y N<br>1 2	

TICK HERE IF CONTINUATION SHEET USED ☐

2A) Just to make sure that I have a complete listing: are there any other persons such as small children or infants that we have not listed? YES ☐ → ADD TO TABLE NO ☐

2B) Are there any other people who may not be members of your family, such as domestic servants, lodgers, or friends who usually live here? YES ☐ → ADD TO TABLE NO ☐

2C) Are there any guests or temporary visitors staying here, or anyone else who stayed here last night, who have not been listed? YES ☐ → ADD TO TABLE NO ☐

CODES FOR Q. 3: RELATIONSHIP TO HEAD OF HOUSEHOLD

01 = HEAD
02 = WIFE OR HUSBAND
03 = SON OR DAUGHTER
04 = SON-IN-LAW OR DAUGHTER-IN-LAW
05 = GRANDCHILD
06 = PARENT
07 = PARENT-IN-LAW
08 = BROTHER OR SISTER
09 = OTHER RELATIVE
10 = ADOPTED/FOSTER/ STEPCHILD
11 = NOT RELATED
99 = DON'T KNOW

Lake Edward/ Lake George HIV Seroprevalence Survey -2015 - Man's questionnaire
UGANDA -KASESE DISTRICT
EPICENTRE

IDENTIFICATION

LANDING SITE ____________________

VILLAGE ____________________

HOUSEHOLD NUMBER . . . . [ ][ ][ ]

CONTACT/PHONE NUMBER ____________________

IDENFICATION NUMBEF . . . . [ ]

INTERVIEWER VISITS

	1	2	3	FINAL VISIT
DATE	________	________	________	DAY [ ][ ]<br>MONTH [ ][ ]<br>YEAR [ ][ ][ ][ ]
INTERVIEWER'S NAME	________	________	________	INT. ID [ ][ ]
RESULT*	________	________	________	RESULT* [ ]
NEXT VISIT: DATE<br>TIME	________<br>________	________<br>________		TOTAL NUMBER OF VISITS [ ]

*RESULT CODES:

1	COMPLETED	4	REFUSED		
2	NOT AT HOME	5	PARTLY COMPLETED	8	OTHER ________ (SPECIFY)
3	POSTPONED	6	INCAPACITATED		

SUPERVISOR	OFFICE EDITOR
NAME ________ [ ]	[ ][ ]

THIS PAGE IS INTENTIONALLY BLANK

C |__| |__||__| HH|__| |__|LN |__| |__|

Lake Edward/ Lake George HIV Seroprevalence Survey -2015 - Men's questionnaire

SECTION 1. RESPONDENT'S BACKGROUND

INTRODUCTION AND CONSENT

NO.	QUESTIONS AND FILTERS	CODING CATEGORIES	SKIP
101	RECORD THE TIME.	HOURS . . . . [ ][ ]<br>MINUTES . . . . [ ][ ]	
102	In what month and year were you born?	MONTH . . . . [ ][ ]<br>DON'T KNOW MONTH . . . . 99<br>YEAR . . . . [ ][ ][ ][ ]<br>DON'T KNOW YEAR 9999	
103	How old are you ?<br>COMPARE AND CORRECT 102 AND/OR 103 IF INCONSISTENT.	AGE IN COMPLETED YEARS [ ][ ]	
104	What is the highest level of school you attended: primary, secondary, or higher?	PRIMARY . . . . 1<br>SECONDARY . . . . 2<br>HIGHER . . . . 3<br>NEVER ATTENDED 4	
105	Where were you born? In Kasese Rubirizi or Kamwenge Districts, in another district in Western Region, in another Region than Western Region or abroad ?	KASESE DISTRIC . . . . 1<br>RUBIRIZI DISTRI . . . . 2<br>KAMWENGE DISTRIC . . . . 3<br>OTHER DISTRICT IN WESTERN REGIOI 4<br>OTHER REGION THAN WESTERN REGI 5<br>OUTSIDE UGANDA 6	
106	How long have you been living continuously at this landing site?<br>IF LESS THAN ONE YEAR, RECORD '00' YEARS AND RECORD MONTHS	YEARS . . . . [ ][ ]<br>MONTHS . . . . [ ][ ]<br>VISITOR . . . . 97<br>ALWAYS 96	 <br>→ 108<br>→ 109
107	Just before you moved here, did you live in Kasese Rubirizi or Kamwenge Districts, in another district in Western Region, in another Region other than Western Region or abroad ?	KASESE DISTRIC . . . . 1<br>RUBIRIZI DISTRI . . . . 2<br>KAMWENGE DISTRIC . . . . 3<br>OTHER DISTRICT IN WESTERN REGIOI 4<br>OTHER REGION THAN WESTERN REGI 5<br>OUTSIDE UGANDA 6	→ 109
108	Where are you currently living?	KASESE DISTRIC . . . . 1<br>RUBIRIZI DISTRI . . . . 2<br>KAMWENGE DISTRIC . . . . 3<br>OTHER DISTRICT IN WESTERN REGIOI 4<br>OTHER REGION THAN WESTERN REGI 5<br>OUTSIDE UGANDA 6	
109	In the last 12 months, how many times have you slept away from home for one or more nights?	NUMBER OF TIMES [ ][ ][ ]<br>NONE 00	 → 112
110	In the last 12 months, have you ever been away from home for more than one month at a time?	YES . . . . 1<br>NO 2	

111	In the last 12 months, when combined, what is the total amount of time you have slept away from this village / landing site?	LESS THAN ONE WEEK .... 1<br>LESS THAN ONE MONTH .... 2<br>LESS THAN THREE MONTH .... 3<br>LESS THAN SIX MONTHS .... 4<br>GREATER THAN SIX MONTHS 5	
112	Are you currently married or living together with a woman as if married?	YES, CURRENTLY MARRIED . . . . 1<br>YES, LIVING WITH A WOMAN . . . . 2<br>NO, NOT IN UNION 3	1, 2 → 115
113	Have you ever been married or lived together with a woman as if married?	YES, FORMERLY MARRIED . . . . 1<br>YES, LIVED WITH A WOMAN . . . . 2<br>NO 3	3 → 115
114	What is your marital status now: are you widowed, divorced, or separated?	WIDOWED . . . . 1<br>DIVORCED . . . . 2<br>SEPARATED 3	
115	How frequently do you use condoms?	NEVER . . . . 1<br>SOMETIMES . . . . 2<br>ALWAYS 3	
116	What kind of work do you do most of the time?	FARMER, FORESTRY . . . . 01<br>FISHING . . . . 02<br>SOLDIER,POLICEMAN . . . . 03<br>SALES, SERVICE WORKER . . . . 04<br>FACTORY WORKER . . . . 05<br>CLERICAL . . . . 06<br>PROFESSIONAL/MANAGER . . . . 07<br>(INCLUDES NURSE, TEACHER)<br>STUDENT . . . . 08<br>HOUSEWIFE . . . . 09<br>NONE . . . . 10<br>OTHER ______________ 98<br>(SPECIFY)	

C |__| |__| |__|HH |__| |__| LN|__| |__|

SECTION 2. CIRCUMCISION

NO.	QUESTIONS AND FILTERS	CODING CATEGORIES	SKIP
201	Some men are circumcised. Are you circumcised?	YES . . . . 1<br>NO . . . . 2<br>DON'T KNOW 9	2, 9 → 205
202	How old were you when you were circumcised?	AGE IN YEARS . . . . [__][__]<br>DURING CHILDHOOD (LESS THAN 5 YEARS OF AGE) . . . 95<br>DON'T KNOW 99	
203	Who circumcised you?	TRADITIONAL PRACTITIONER . . . . . 1<br>FAMILY/FRIEND . . . . 2<br>HEALTH WORKER/ HEALTH PROFESSIONAL . . . . 3<br>RELIGIOUS LEADER . . . . 4<br>OTHER . . . . 8<br>DON'T KNOW 9	
204	Where did you go to be circumcised?	HOSPITAL /CLINIC/MMC CENTER. . . . . 1<br>HOME OF A HEALTH WORKER/ HEALTH PROFESSIONAL ..... 2<br>OWN HOME . . . . 3<br>OTHER HOME . . . . 4<br>OTHER . . . . 8<br>DON'T KNOW 9	
205	Do you think circumcision can prevent the transmission of HIV?	YES . . . . 1<br>NO . . . . 2<br>DON'T KNOW 9	

C |__| |__| |__| HH|__| |__|LN |__| |__|

SECTION 4. HIV/AIDS

NO.	QUESTIONS AND FILTERS	CODING CATEGORIES	SKIP
401	Now I would like to talk about something else. Have you ever heard of an illness called AIDS?	YES . . . . . 1<br>NO 2	 → 413
402	Can the virus that causes AIDS be transmitted from a mother to her baby?	YES NO DK<br>IN GENERAL 1 2 9	If NO or DK<br>→ 403
	Can the virus that causes AIDS be transmitted from a mother to her baby:<br>During pregnancy?<br>During delivery?<br>By breastfeeding?	 <br>DURING PREG. . . . . . . 1 2 9<br>DURING DELIVERY . . . . 1 2 9<br>BREASTFEEDING 1 2 9	
403	Are there any special drugs that a doctor or a nurse can give to a woman infected with the AIDS virus to reduce the risk of transmission to the baby?	YES . . . . . 1<br>NO . . . . . 2<br>DON'T KNOW 9	
404	Do you know of a place where people can go to get tested for the AIDS virus?	YES . . . . . 1<br>NO 2	 → 406
405	Where is that place?<br><br>Any other place?<br><br>PROBE TO IDENTIFY EACH TYPE OF SOURCE.<br><br>IF UNABLE TO DETERMINE IF PUBLIC OR PRIVATE SECTOR, WRITE THE NAME OF THE PLACE<br><br>______________________<br>(NAME OF PLACE(S))	YES NO<br>*PUBLIC SECTOR*<br>GOVERNMENT HOSPITAL . . . 11 0<br>GOVT. HEALTH CENTER. . . . . . 12 0<br>STAND-ALONE VCT CENTER 13 0<br>FAMILY PLANNING CLINIC. . . . . 14 0<br>MOBILE CLINIC . . . . 15 0<br>FIELDWORKER . . . . 16 0<br>SCHOOL BASED CLINIC. . . . . . . 17 0<br>OTHER PUBLIC SECTOR 18 0<br>______________________<br>(IF 'OTHER' SPECIFY)<br><br>*PRIVATE MEDICAL SECTOR*<br>PRIVATE HOSPITAL/CLINIC/<br>PRIVATE DOCTOR . . . . 21 0<br>STAND-ALONE VCT CENTER . . . . 22 0<br>PHARMACY . . . . 23 0<br>MOBILE CLINIC . . . . 24 0<br>FIELDWORKER . . . . 25 0<br>OTHER PRIVATE SECTOR . . 28 0<br>______________________<br>(IF 'OTHER' SPECIFY)<br><br>OTHER ______________________ 98 0<br>(SPECIFY)	
406	Now I would like to ask you some questions about your own experience of HIV testing. Your answers are completely private. This form will not have your name anywhere on it; instead you will be identified by a number		
407	Have you ever been tested to see if you have the AIDS virus?	YES . . . . . 1<br>NO 2	 → 413
408	How many times have you had an HIV test in your lifetime?	NUMBER OF TIMES . . . . [ ][ ]<br>DON'T KNOW 99	

NO.	QUESTIONS AND FILTERS	CODING CATEGORIES	SKIP
409	How many months ago was your most recent HIV test?	MONTHS AGO . . . . [ ][ ]<br>TWO OR MORE YEARS 95	
410	Where was the test done?<br><br>PROBE TO IDENTIFY THE TYPE OF SOURCE.<br><br>IF UNABLE TO DETERMINE IF PUBLIC OR PRIVATE SECTOR WRITE THE NAME OF THE PLACE<br><br>______________________<br>(NAME OF PLACE)	*PUBLIC SECTOR*<br>GOVERNMENT HOSPITAL . . . 11<br>GOVT. HEALTH CENTER. . . . . . 12<br>STAND-ALONE VCT CENTER 13<br>FAMILY PLANNING CLINIC. . . . . 14<br>MOBILE CLINIC . . . . 15<br>FIELDWORKER . . . . 16<br>SCHOOL BASED CLINIC. . . . . . . 17<br>OTHER PUBLIC SECTOR 18<br>______________________<br>(IF 'OTHER' SPECIFY)<br><br>*PRIVATE MEDICAL SECTOR*<br>PRIVATE HOSPITAL/CLINIC/<br>PRIVATE DOCTOR . . . . 21<br>STAND-ALONE VCT CENTER 22<br>PHARMACY . . . . 23<br>MOBILE CLINIC . . . . 24<br>FIELDWORKER . . . . 25<br>OTHER PRIVATE SECTOR 28<br>______________________<br>(IF 'OTHER' SPECIFY)<br><br>*OTHER SOURCE*<br>HOME . . . . 31<br>CORRECTIONAL FACILITY . . . . 32<br><br>OTHER ______________________ 98<br>(SPECIFY)	
411	Did you get the results of the test?	YES . . . . 1<br>NO 2	 → 413
412	I would like to ask you the result of your latest HIV test, but I want to remind you again that you should only answer the question if you feel comfortable. If you feel comfortable, could you tell me the result of your latest HIV test?	POSITIVE . . . . 1<br>NEGATIVE . . . . 2<br>INDETERMINATE . . . . 3<br>REFUSE TO ANSWER . . . . 4<br>DON'T KNOW 9	
413	THANK THE PATIENT FOR HIS PARTICIPATION<br><br>START PRE-COUNSELLING		

SECTION 6. ART Coverage

NO.	QUESTIONS AND FILTERS	CODING CATEGORIES	SKIP
	We are going to talk now about your knowledge and care and treatment for HIV. In order for MSF to improve the services provided it is very important to answer in the truest way possible, there will be no jugement, and no consequences.		
601	Did you know you were infected with AIDS?	YES . . . . . 1<br>NO 2	
602	Have you already had a HIV test that showed you were infected with AIDS?	YES . . . . . 1<br>NO 2	
603	CHECK 502:<br>KNEW HIV STATUS ☐ ↓	NEW PATIENT ☐	→ END
	We are now going to talk about care and treatment. We know they are many reasons which could lead the patient to wisthdraw from HIV care. Again it is very important to answer in the truest way possible, there will be no jugement, and no consequences		
604	When were you first tested positive for the AIDS virus?	MONTH . . . . . [ ][ ]<br>DON'T KNOW . . . . . 99<br>YEAR . . . . . [ ][ ][ ][ ]<br>DON'T KNOW 9999	
605	Where was this test done?<br><br>PROBE TO IDENTIFY THE TYPE OF SOURCE.<br><br>IF UNABLE TO DETERMINE IF PUBLIC OR PRIVATE SECTOR, WRITE THE NAME OF THE PLACE.<br><br>__________<br>(NAME OF PLACE)	*PUBLIC SECTOR*<br>GOVERNMENT HOSPITAL . . . . . 11<br>GOVT. HEALTH CENTER . . . . . 12<br>STAND-ALONE VCT CENTER . . . 13<br>FAMILY PLANNING CLINIC . . . . . 14<br>MOBILE CLINIC . . . . . 15<br>FIELDWORKER . . . . . 16<br>SCHOOL BASED CLINIC . . . . . 17<br>OTHER PUBLIC SECTOR _____ 18<br>(SPECIFY)<br>*PRIVATE MEDICAL SECTOR*<br>PRIVATE HOSPITAL/CLINIC/ PRIVATE DOCTOR . . . . . 21<br>STAND-ALONE VCT CENTER . . . . . 22<br>PHARMACY . . . . . 23<br>MOBILE CLINIC . . . . . 24<br>FIELDWORKER . . . . . 25<br>OTHER PRIVATE MEDICAL SECTOR _____ 28<br>(SPECIFY)<br>*OTHER SOURCE*<br>HOME . . . . . 31<br>CORRECTIONAL FACILITY . . . . . 32<br>OTHER _____ 98<br>(SPECIFY)	
606	After you discovered you were infected with the virus that causes AIDS, did you ever seek care for the AIDS infection?	YES . . . . . 1<br>NO 2	 → END

NO.	QUESTIONS AND FILTERS	CODING CATEGORIES	SKIP
607	Did you get some blood sample taken to check the CD4 when you first went to receive care for the AIDS virus?	YES . . . . . 1<br>NO . . . . . 2<br>DK 9	(NO, DK) → 609
608	Did you get the results of this exam?	YES . . . . . 1<br>NO . . . . . 2<br>DK 9	
609	Have you ever initiated ART, Antiretroviral Treatment, drugs against the AIDS virus?	YES . . . . . 1<br>NO 2	(NO) → 616
610	When did you first start Antiretroviral therapy?<br><br>CHECK HEALTH BOOKLET	MONTH . . . . . [ ][ ]<br>DON'T KNOW . . . . . 99<br><br>YEAR . . . . . [ ][ ][ ][ ]<br>DON'T KNOW 9999	
611	Are you still receiving ART, Antiretroviral Treatment, drugs against the AIDS virus?<br><br>CHECK THE DRUGS AND INSURE THEY ARE ARVs	YES . . . . . 1<br>NO 2	(NO) → 621
612	When was your last consultation?<br><br>CHECK HEALTH BOOKLET	MONTH . . . . . [ ][ ]<br>DON'T KNOW . . . . . 99<br><br>YEAR . . . . . [ ][ ][ ][ ]<br>DON'T KNOW 9999	
613	Where are you now receiving ART?<br><br>PROBE TO IDENTIFY THE TYPE OF SOURCE.<br><br>IF UNABLE TO DETERMINE IF PUBLIC OR PRIVATE SECTOR, WRITE THE NAME OF THE PLACE.<br><br>________________<br>(NAME OF PLACE)	*PUBLIC SECTOR*<br>GOVERNMENT HOSPITAL . . . . . 11<br>GOVT. HEALTH CENTER . . . . . 12<br>GOVT. DISPENSARY . . . . . 13<br><br>OTHER PUBLIC SECTOR ________ 18<br>(SPECIFY)<br><br>*PRIVATE MEDICAL SECTOR*<br>PRIVATE HOSPITAL/CLINIC/ PRIVATE DOCTOR . . . . . 21<br><br>OTHER PRIVATE SECTOR ________ 28<br>(SPECIFY)<br>OTHER ________ 98<br>(SPECIFY)	
614	In which district is this place?	KASESE DISTRIC . . . . . 1<br>RUBIRIZI DISTRI . . . . . 2<br>KAMWENGE DISTRIC . . . . . 3<br>OTHER ________ 8<br>(SPECIFY)	

615	What is the name of this place?<br><br>SEE CODES OF FACILITIES IN ANNEX	______ (SPECIFY) [ ][ ]	→ END
616	Are you still followed up for the AIDS infection?	YES . . . . 1<br>NO 2	 → 621
617	When was your last consultation?<br><br>(IF NEEDED CHECK ON THE HEALTH BOOKLET)	MONTH . . . . [ ][ ]<br>DON'T KNOW . . . . 99<br>YEAR . . . . . . [ ][ ][ ][ ]<br>DON'T KNOW 9999	
618	Where are you now receiving care?<br><br>PROBE TO IDENTIFY THE TYPE OF SOURCE.<br><br>IF UNABLE TO DETERMINE IF PUBLIC OR PRIVATE SECTOR, WRITE THE NAME OF THE PLACE.<br><br>______<br>(NAME OF PLACE)	*PUBLIC SECTOR*<br>GOVERNMENT HOSPITAL . . . . 11<br>GOVT. HEALTH CENTER . . . . 12<br><br>OTHER PUBLIC SECTOR ______ 18<br>(SPECIFY)<br><br>*PRIVATE MEDICAL SECTOR*<br>PRIVATE HOSPITAL/CLINIC/ PRIVATE DOCTOR . . . . 21<br><br>OTHER PRIVATE MEDICAL SECTOR ______ 28<br>(SPECIFY)<br><br>OTHER ______ 98<br>(SPECIFY)	
619	In which district is this place?	KASESE DISTRIC . . . . 1<br>RUBIRIZI DISTRI . . . . 2<br>KAMWENGE DISTRIC . . . . 3<br>OTHER ______ 8<br>(SPECIFY)	
620	What was the name of this place?<br><br>SEE CODES OF FACILITIES IN ANNEX	______ (SPECIFY) [ ][ ]	→ END
621	Why did you stop?	NO ONE WAS ATTENDING 1<br>STOCK OUT 2<br>UNFRIENDLY STAFF 3<br>SPONTANEOUS 4<br>ADVISED TO STOP 5<br>THOUGHT I WAS CURED/ FEEL GOOD 6<br>SIDE EFFECT 7<br>MOVED AWAY 8<br>TRANSPORT COST 9<br>STOP PMTCT 10<br><br>OTHER ______ 98<br>(SPECIFY)	

INTERVIEWER'S OBSERVATIONS

TO BE FILLED IN AFTER COMPLETING INTERVIEW

COMMENTS ABOUT RESPONDENT:

COMMENTS ON SPECIFIC QUESTIONS:

ANY OTHER COMMENTS:

SUPERVISOR'S OBSERVATIONS

NAME OF SUPERVISOR: ______________________ DATE: ______________________

EDITOR'S OBSERVATIONS

NAME OF EDITOR: ______________________ DATE: ______________________

IDENTIFICATION

LANDING SITE ____________________

VILLAGE ____________________

HOUSEHOLD NUMBER . . . . [ ][ ][ ]

CONTACT/PHONE NUMBER ____________________

IDENFICATION NUMBEF . . . .

INTERVIEWER VISITS

	1	2	3	FINAL VISIT
DATE	______	______	______	DAY [ ][ ]<br>MONTH [ ][ ]<br>YEAR [ ][ ][ ][ ]
INTERVIEWER'S NAME	______	______	______	INT. ID [ ][ ]
RESULT*	______	______	______	RESULT* [ ]
NEXT VISIT: DATE<br>TIME	______<br>______	______<br>______		TOTAL NUMBER OF VISITS [ ]

*RESULT CODES:

1	COMPLETED	4	REFUSED		
2	NOT AT HOME	5	PARTLY COMPLETED	8	OTHER ____________
3	POSTPONED	6	INCAPACITATED		(SPECIFY)

SUPERVISOR	OFFICE EDITOR
NAME ____________ [ ]	[ ][ ]

THIS PAGE IS INTENTIONALLY BLANK

C |__| |__| |__| HH|__| |__|LN |__| |__|

Lake Edward/ Lake George HIV Seroprevalence Survey -2015 - Women's questionnaire

SECTION 1. RESPONDENT'S BACKGROUND

INTRODUCTION AND CONSENT

NO.	QUESTIONS AND FILTERS	CODING CATEGORIES	SKIP
101	RECORD THE TIME.	HOURS . . . . [ ][ ]<br>MINUTES . . . . [ ][ ]	
102	In what month and year were you born?	MONTH . . . . [ ][ ]<br>DON'T KNOW MONTH . . . . 99<br>YEAR . . . . [ ][ ][ ][ ]<br>DON'T KNOW YEAR 9999	
103	How old are you ?<br>COMPARE AND CORRECT 102 AND/OR 103 IF INCONSISTENT.	AGE IN COMPLETED YEARS [ ][ ]	
104	What is the highest level of school you attended: primary, secondary, or higher?	PRIMARY . . . . 1<br>SECONDARY . . . . 2<br>HIGHER . . . . 3<br>NEVER ATTENDED 4	
105	Where were you born? In Kasese Rubirizi or Kamwenge Districts, in another district in Western Region, in another Region than Western Region or abroad ?	KASESE DISTRIC . . . . 1<br>RUBIRIZI DISTRIC . . . . 2<br>KAMWENGE DISTRICT . . . . 3<br>OTHER DISTRICT IN WESTERN REGION 2<br>OTHER REGION THAN WESTERN REGI 3<br>OUTSIDE UGANDA 4	
106	How long have you been living continuously at this landing site?<br>IF LESS THAN ONE YEAR, RECORD '00' YEARS AND RECORD MONTHS	YEARS . . . . [ ][ ]<br>MONTHS . . . . [ ][ ]<br>VISITOR . . . . 97<br>ALWAYS 96	 <br>→ 108<br>→ 109
107	Just before you moved here, did you live in Kasese Rubirizi or Kamwenge Districts, in another district in Western Region, in another Region other than Western Region or abroad ?	KASESE DISTRIC . . . . 1<br>RUBIRIZI DISTRIC . . . . 2<br>KAMWENGE DISTRICT . . . . 3<br>OTHER DISTRICT IN WESTERN REGION 2<br>OTHER REGION THAN WESTERN REGI 3<br>OUTSIDE UGANDA 4	→ 109
108	Where are you currently living?	KASESE DISTRIC . . . . 1<br>RUBIRIZI DISTRIC . . . . 2<br>KAMWENGE DISTRICT . . . . 3<br>OTHER DISTRICT IN WESTERN REGION 2<br>OTHER REGION THAN WESTERN REGI 3<br>OUTSIDE UGANDA 4	
109	In the last 12 months, how many times have you slept away from home for one or more nights?	NUMBER OF TIMES [ ][ ][ ]<br>NONE 00	 → 112
110	In the last 12 months, have you been away from home for more than one month at a time?	YES . . . . 1<br>NO 2	

111	In the last 12 months, when combined, what is the total amount of time you have slept away from this village / landing site?	LESS THAN ONE WEEK .... 1<br>LESS THAN ONE MONTH .... 2<br>LESS THAN THREE MONTH .... 3<br>LESS THAN SIX MONTHS .... 4<br>GREATER THAN SIX MONTHS 5	
112	Are you currently married or living together with a man as if married?	YES, CURRENTLY MARRIED . . . . 1<br>YES, LIVING WITH A MAN . . . . 2<br>NO, NOT IN UNION 3	115
113	Have you ever been married or lived together with a man as if married?	YES, FORMERLY MARRIED . . . . 1<br>YES, LIVED WITH A MAN . . . . 2<br>NO 3	→ 115
114	What is your marital status now: are you widowed, divorced, or separated?	WIDOWED . . . . 1<br>DIVORCED . . . . 2<br>SEPARATED 3	
115	How frequently do you use condoms?	NEVER . . . . 1<br>SOMETIMES . . . . 2<br>ALWAYS 3	
116	What kind of work do you do most of the time?	FARMER, FORESTRY . . . . 01<br>FISHING . . . . 02<br>SOLDIER,POLICEMAN . . . . 03<br>SALES, SERVICE WORKER . . . . 04<br>FACTORY WORKER . . . . 05<br>CLERICAL . . . . 06<br>PROFESSIONAL/MANAGER . . . . 07<br>(INCLUDES NURSE, TEACHER)<br>STUDENT . . . . 08<br>HOUSEWIFE . . . . 09<br>NONE . . . . 10<br>OTHER ________________ 98<br>(SPECIFY)	

C |__| |__| |__|HH |__| |__|LN |__| |__|

SECTION 2. REPRODUCTION

NO.	QUESTIONS AND FILTERS	CODING CATEGORIES	SKIP
201	Now I would like to ask about all the births you have had during your life. Have you ever given birth?	YES . . . . . 1<br>NO 2	 → 206
202	Do you have any sons or daughters to whom you have given birth who are now living with you?	YES . . . . . 1<br>NO 2	 → 204
203	How many sons live with you?<br>And how many daughters live with you?<br>IF NONE, RECORD '00'.	SONS AT HOME . . . . . [ ][ ]<br>DAUGHTERS AT HOME . . . . . [ ][ ]	
204	Do you have any sons or daughters to whom you have given birth who are alive but do not live with you?	YES . . . . . 1<br>NO 2	 → 206
205	How many sons are alive but do not live with you?<br>And how many daughters are alive but do not live with you?<br>IF NONE, RECORD '00'.	SONS ELSEWHERE . . . . . [ ][ ]<br>DAUGHTERS ELSEWHERE [ ][ ]	
206	Have you ever given birth to a boy or girl who was born alive but later died?<br>IF NO, PROBE: Any baby who cried or showed signs of life but did not survive?	YES . . . . . 1<br>NO 2	 → 208
207	How many boys have died?<br>And how many girls have died?<br>IF NONE, RECORD '00'.	BOYS DEAD . . . . . [ ][ ]<br>GIRLS DEAD . . . . . [ ][ ]	
208	SUM ANSWERS TO 203, 205, AND 207, AND ENTER TOTAL.<br>IF NONE, RECORD '00'.	TOTAL BIRTHS . . . . . [ ][ ]	
209	CHECK 208:<br>Just to make sure that I have this right: you have had in TOTAL _____ births during your life. Is that correct?<br>YES [ ] ↓ NO [ ] → PROBE AND CORRECT 201-208 AS NECESSARY.	**IF '0' BIRTH → 211**	
210	In what month and year was your last child born?	MONTHS [ ][ ]<br>YEAR [ ][ ][ ][ ]<br>DON'T KNOW . 9999	
211	Are you currently pregnant?	YES . . . . . 1<br>NO . . . . . 2<br>DK 9	 NO, DK → 301
	IF YES, PROBE: How many months pregnant are you?	MONTHS . . . . . [ ]	

Lake Edward/ Lake George HIV Seroprevalence Survey -2015 - Women's questionnaire

SECTION 3. PREGNANCY

301	CHECK 210: ONE OR MORE BIRTHS IN 2011 OR LATER ☐ ↓ / NO BIRTHS IN 2011 OR LATER ☐	→ 401

NO.	QUESTIONS AND FILTERS	CODING CATEGORIES	SKIP
302	What name was given to your last baby?	NAME ____________________	
303	Is (NAME) still alive?	YES . . . 1<br>NO . . . 2	
304	Did you see anyone for antenatal care for this pregnancy?	YES . . . 1<br>NO . . . 2	→ 309
305	Whom did you see?<br><br>Anyone else?<br><br>PROBE TO IDENTIFY EACH TYPE OF PERSON AND RECORD ALL MENTIONED.	YES NO<br>*HEALTH PERSONNEL*<br>DOCTOR . . . 1 0<br>NURSE/MIDWIFE . . . 2 0<br>AUXILIARY MIDWIFE . . . 3 0<br>*OTHER PERSON*<br>TRADITIONAL BIRTH ATTENDANT . . . 4 0<br>COMMUNITY HEALTH WORKER . . . 5 0<br>OTHER . . . 8 0<br>____________<br>SPECIFY	
306	Where did you receive antenatal care for this pregnancy?<br><br>Anywhere else?<br><br>PROBE TO IDENTIFY EACH TYPE OF SOURCE.<br><br>IF UNABLE TO DETERMINE IF PUBLIC OR PRIVATE SECTOR, WRITE THE NAME OF THE PLACE<br>____________________<br>(NAME OF PLACE(S))	YES NO<br>*HOME*<br>YOUR HOME . . . 31 0<br>OTHER HOME . . . 32 0<br><br>*PUBLIC SECTOR*<br>GOVT. HOSPITAL . . . 11 0<br>GOVT. HEALTH CENTER . . 12 0<br>OTHER PUBLIC SECTOR . . 18 0<br>____________<br>(SPECIFY)<br>*PRIVATE MED. SECTOR*<br>PVT. HOSPITAL/CLINIC . . 21 0<br>OTHER PRIVATE SECTOR 28 0<br>____________<br>(SPECIFY)<br>OTHER . . . 98 0<br>____________<br>(SPECIFY)	
307	How many months pregnant were you when you first received antenatal care for this pregnancy?	MONTHS . . . [ ][ ]<br>DON'T KNOW . . . 99	
308	How many times did you receive antenatal care during this pregnancy?	NUMBER OF TIMES . . . [ ][ ]<br>DON'T KNOW . . . 99	

NO	QUESTIONS AND FILTERS	LAST BIRTH<br>NAME ________	SKIP
309	Who assisted with the delivery of (NAME)?<br><br>Anyone else?<br>PROBE FOR THE TYPE(S) OF PERSON(S) AND RECORD ALL MENTIONED<br><br>IF RESPONDENT SAYS NO ONE ASSISTED PROBE TO DETERMINE WETHER ANY ADULTS WERE PRESENT AT DELIVERY	YES / NO<br>*HEALTH PERSONNEL*<br>DOCTOR . . . . . . 1 / 0<br>NURSE/MIDWIFE 2 / 0<br>AUXILIARY MIDWIFE 3 / 0<br>*OTHER PERSON*<br>TRADITIONAL BIRTH ATTENDANT . . 4 / 0<br>RELATIVE/FRIEND . 5 / 0<br>OTHER ________ (SPECIFY) 8 / 0<br>NO ONE ASSISTED 9 / 0	
310	Where did you give birth to (NAME)?<br><br>PROBE TO IDENTIFY THE TYPE OF SOURCE.<br><br>IF UNABLE TO DETERMINE IF PUBLIC OR PRIVATE SECTOR, WRITE THE NAME OF THE PLACE.<br><br>________ (NAME OF PLACE)	*HOME*<br>YOUR HOME . . . . 31<br>OTHER HOME . . . . 32<br>*PUBLIC SECTOR*<br>GOVT. HOSPITAL . . . . 11<br>GOVT. HEALTH CENTER 12<br>OTHER PUBLIC SECTOR 18<br>________ (SPECIFY)<br>*PRIVATE MED. SECTOR*<br>PVT. HOSPITAL/CLINIC . . 21<br>OTHER PRIVATE SECTOR 28<br>________ (SPECIFY)<br>OTHER . . . . 98<br>________ (SPECIFY)	31 → 312<br><br>98 → 312
311	How long after (NAME) was delivered did you stay there?<br><br>IF LESS THAN ONE DAY, RECORD HOURS<br>IF LESS THAN ONE WEEK,RECORD DAYS	HOURS 1 . . . . . . [ ][ ]<br>DAYS 2 . . . . . . [ ][ ]<br>WEEKS 3 . . . . . . [ ][ ]<br>DON'T KNOW 99	
312	Did you ever breastfeed (NAME)?	YES . . . . 1<br>NO 2	2 → 401
313	CHECK 303: IS THE CHILD LIVING?	LIVING [ ] ↓ DEAD [ ]	DEAD → 401
314	Are you still breastfeeding (NAME)?	YES . . . . 1<br>NO 2	1 → 401
315	What age was (NAME) when you stopped to breasfeed him/her?	MONTHS . . . . [ ][ ]<br>DON'T KNOW 99	

SECTION 4. HIV/AIDS

NO.	QUESTIONS AND FILTERS	CODING CATEGORIES	SKIP
401	Now I would like to talk about something else. Have you ever heard of an illness called AIDS?	YES . . . 1<br>NO . . . 2	 → 421
402	Can the virus that causes AIDS be transmitted from a mother to her baby?	YES / NO / DK<br>IN GENERAL . . . 1 / 2 / 9	If NO or DK → 403
	Can the virus that causes AIDS be transmitted from a mother to her baby:<br>During pregnancy?<br>During delivery?<br>By breastfeeding?	YES / NO / DK<br>DURING PREG. . . . 1 / 2 / 9<br>DURING DELIVERY . . . 1 / 2 / 9<br>BREASTFEEDING . . . 1 / 2 / 9	
403	Are there any special drugs that a doctor or a nurse can give to a woman infected with the AIDS virus to reduce the risk of transmission to the baby?	YES . . . 1<br>NO . . . 2<br>DON'T KNOW . . . 9	
404	Do you know of a place where people can go to get tested for the AIDS virus?	YES . . . 1<br>NO . . . 2	 → 406
405	Where is that place?<br><br>Any other place?<br><br>PROBE TO IDENTIFY EACH TYPE OF SOURCE.<br><br>IF UNABLE TO DETERMINE IF PUBLIC OR PRIVATE SECTOR, WRITE THE NAME OF THE PLACE<br><br>__________<br>(NAME OF PLACE(S))	YES / NO<br>*PUBLIC SECTOR*<br>GOVERNMENT HOSPITAL . . . 11 / 0<br>GOVT. HEALTH CENTER. . . . 12 / 0<br>STAND-ALONE VCT CENTER 13 / 0<br>FAMILY PLANNING CLINIC. . . . 14 / 0<br>MOBILE CLINIC . . . 15 / 0<br>FIELDWORKER . . . 16 / 0<br>SCHOOL BASED CLINIC. . . . 17 / 0<br>OTHER PUBLIC SECTOR 18 / 0<br>__________<br>(IF 'OTHER' SPECIFY)<br><br>*PRIVATE MEDICAL SECTOR*<br>PRIVATE HOSPITAL/CLINIC/ PRIVATE DOCTOR . . . 21 / 0<br>STAND-ALONE VCT CENTER . . . 22 / 0<br>PHARMACY . . . 23 / 0<br>MOBILE CLINIC . . . 24 / 0<br>FIELDWORKER . . . 25 / 0<br>OTHER PRIVATE SECTOR . . 28 / 0<br>__________<br>(IF 'OTHER' SPECIFY)<br><br>OTHER __________ 98 / 0<br>(SPECIFY)	
406	Now I would like to ask you some questions about your own experience of HIV testing. Your answers are completely private. This form will not have your name anywhere on it; instead you will be identified by a number		
407	Have you ever been tested to see if you have the AIDS virus?	YES . . . 1<br>NO . . . 2	 → 421
408	How many times have you had an HIV test in your lifetime?	NUMBER OF TIMES . . . [ ][ ]<br>DON'T KNOW . . . 99	

NO.	QUESTIONS AND FILTERS	CODING CATEGORIES	SKIP
409	How many months ago was your most recent HIV test?	MONTHS AGO . . . . |__|__| TWO OR MORE YEARS 95	
410	Where was the test done?<br><br>PROBE TO IDENTIFY THE TYPE OF SOURCE.<br><br>IF UNABLE TO DETERMINE IF PUBLIC OR PRIVATE SECTOR WRITE THE NAME OF THE PLACE<br><br>_______________<br>(NAME OF PLACE)	*PUBLIC SECTOR*<br>GOVERNMENT HOSPITAL . . . 11<br>GOVT. HEALTH CENTER. . . . . . 12<br>STAND-ALONE VCT CENTER 13<br>FAMILY PLANNING CLINIC. . . . . 14<br>MOBILE CLINIC . . . . 15<br>FIELDWORKER . . . . 16<br>SCHOOL BASED CLINIC. . . . . . . 17<br>OTHER PUBLIC SECTOR 18<br>_______________<br>(IF 'OTHER' SPECIFY)<br><br>*PRIVATE MEDICAL SECTOR*<br>PRIVATE HOSPITAL/CLINIC/<br>PRIVATE DOCTOR . . . . 21<br>STAND-ALONE VCT CENTER 22<br>PHARMACY . . . . 23<br>MOBILE CLINIC . . . . 24<br>FIELDWORKER . . . . 25<br>OTHER PRIVATE SECTOR 28<br>_______________<br>(IF 'OTHER' SPECIFY)<br><br>*OTHER SOURCE*<br>HOME . . . . 31<br>CORRECTIONAL FACILITY . . . . 32<br><br>OTHER _______________ 98<br>(SPECIFY)	
411	Did you get the results of the test?	YES . . . . 1<br>NO 2	 → 413
412	I would like to ask you the result of your latest HIV test, but I want to remind you again that you should only answer the question if you feel comfortable. If you feel comfortable, could you tell me the result of your latest HIV test?	POSITIVE . . . . 1<br>NEGATIVE . . . . 2<br>INDETERMINATE . . . . 3<br>REFUSE TO ANSWER . . . . 4<br>DON'T KNOW 9	

NO.	QUESTIONS AND FILTERS	CODING CATEGORIES	SKIP
413	CHECK 208: BIRTH(S) ☐ ↓	NO BIRTHS ☐	→ 421
414	CHECK 210<br>LAST BIRTH DURING OR AFTER 2013 ☐ ↓	LAST BIRTH BEFORE 2013 ☐	→ 421
415	CHECK 304 FOR LAST BIRTH:<br>HAD ANTENATAL CARE ☐ ↓	NO ANTENATAL CARE ☐	→ 421
416	Were you offered a test for the AIDS virus as part of your antenatal care?	YES . . . . . 1<br>NO 2	 → 421
417	Were you tested for the AIDS virus as part of your antenatal care?	YES . . . . . 1<br>NO 2	 → 421
418	How many months pregnant were you when you were first tested for the AIDS virus as a part of your antenatal care for this pregnancy?	MONTHS . . . . . [ ][ ]<br>DON'T KNOW 99	
419	Where was the test done?<br><br>PROBE TO IDENTIFY THE TYPE OF SOURCE.<br><br>IF UNABLE TO DETERMINE IF PUBLIC OR PRIVATE SECTOR, WRITE THE NAME OF THE PLACE<br><br>____________<br>(NAME OF PLACE)	*PUBLIC SECTOR*<br>GOVERNMENT HOSPITAL . . . 11<br>GOVT. HEALTH CENTER. . . . . . 12<br>STAND-ALONE VCT CENTER 13<br>FAMILY PLANNING CLINIC. . . . . 14<br>MOBILE CLINIC . . . . 15<br>FIELDWORKER . . . . 16<br>SCHOOL BASED CLINIC. . . . . . . 17<br>OTHER PUBLIC SECTOR 18<br>____________<br>(IF 'OTHER' SPECIFY)<br><br>*PRIVATE MEDICAL SECTOR*<br>PRIVATE HOSPITAL/CLINIC/<br>PRIVATE DOCTOR . . . . 21<br>STAND-ALONE VCT CENTER 22<br>PHARMACY . . . . 23<br>MOBILE CLINIC . . . . 24<br>FIELDWORKER . . . . 25<br>OTHER PRIVATE SECTOR 28<br>____________<br>(IF 'OTHER' SPECIFY)<br><br>*OTHER SOURCE*<br>HOME . . . . 31<br>CORRECTIONAL FACILITY . . . . 32<br><br>OTHER ____________ 98<br>(SPECIFY)	
420	Did you get the results of the test?	YES . . . . . 1<br>NO 2	
421	THANK THE PATIENT FOR HER PARTICIPATION<br><br>START PRE-COUNSELLING		

SECTION 6. ART Coverage

NO.	QUESTIONS AND FILTERS	CODING CATEGORIES	SKIP
	We are going to talk now about your knowledge and care and treatment for HIV. In order for MSF to improve the services provided it is very important to answer in the truest way possible, there will be no jugement, and no consequences.		
601	Did you know you were infected with AIDS?	YES . . . . . 1<br>NO 2	
602	Have you already had a HIV test that showed you were infected with AIDS?	YES . . . . . 1<br>NO 2	
603	CHECK 502:<br>KNEW HIV STATUS ☐ ↓	NEW PATIENT ☐	→ END
	We are now going to talk about care and treatment. We know they are many reasons which could lead the patient to wisthdraw from HIV care. Again it is very important to answer in the truest way possible, there will be no jugement, and no consequences		
604	When were you first tested positive for the AIDS virus?	MONTH . . . . . [ ][ ]<br>DON'T KNOW . . . . . 99<br>YEAR . . . . . [ ][ ][ ][ ]<br>DON'T KNOW 9999	
605	Where was this test done?<br><br>PROBE TO IDENTIFY THE TYPE OF SOURCE.<br><br>IF UNABLE TO DETERMINE IF PUBLIC OR PRIVATE SECTOR, WRITE THE NAME OF THE PLACE.<br><br>______________________<br>(NAME OF PLACE)	*PUBLIC SECTOR*<br>GOVERNMENT HOSPITAL . . . . . 11<br>GOVT. HEALTH CENTER . . . . . 12<br>STAND-ALONE VCT CENTER . . . 13<br>FAMILY PLANNING CLINIC . . . . . 14<br>MOBILE CLINIC . . . . . 15<br>FIELDWORKER . . . . . 16<br>SCHOOL BASED CLINIC . . . . . 17<br>OTHER PUBLIC SECTOR ________ 18<br>(SPECIFY)<br>*PRIVATE MEDICAL SECTOR*<br>PRIVATE HOSPITAL/CLINIC/ PRIVATE DOCTOR . . . . . 21<br>STAND-ALONE VCT CENTER . . . . . 22<br>PHARMACY . . . . . 23<br>MOBILE CLINIC . . . . . 24<br>FIELDWORKER . . . . . 25<br>OTHER PRIVATE MEDICAL SECTOR ________ 28<br>(SPECIFY)<br><br>*OTHER SOURCE*<br>HOME . . . . . 31<br>CORRECTIONAL FACILITY . . . . . 32<br><br>OTHER ________ 98<br>(SPECIFY)	
606	After you discovered you were infected with the virus that causes AIDS, did you ever seek care for the AIDS infection?	YES . . . . . 1<br>NO 2	 NO → END

NO.	QUESTIONS AND FILTERS	CODING CATEGORIES	SKIP
607	Did you get some blood sample taken to check the CD4 when you first went to receive care for the AIDS virus?	YES . . . . . 1<br>NO . . . . . 2<br>DK 9	NO, DK → 609
608	Did you get the results of this exam?	YES . . . . . 1<br>NO . . . . . 2<br>DK 9	
609	Have you ever initiated ART, Antiretroviral Treatment, drugs against the AIDS virus?	YES . . . . . 1<br>NO 2	NO → 616
610	When did you first start Antiretroviral therapy?<br><br>CHECK HEALTH BOOKLET	MONTH . . . . . [ ][ ]<br>DON'T KNOW . . . . . 99<br>YEAR . . . . . [ ][ ][ ][ ]<br>DON'T KNOW 9999	
611	Are you still receiving ART, Antiretroviral Treatment, drugs against the AIDS virus?<br><br>CHECK THE DRUGS AND INSURE THEY ARE ARVs	YES . . . . . 1<br>NO 2	NO → 621
612	When was your last consultation?<br><br>CHECK HEALTH BOOKLET	MONTH . . . . . [ ][ ]<br>DON'T KNOW . . . . . 99<br>YEAR . . . . . [ ][ ][ ][ ]<br>DON'T KNOW 9999	
613	Where are you now receiving ART?<br><br>PROBE TO IDENTIFY THE TYPE OF SOURCE.<br><br>IF UNABLE TO DETERMINE IF PUBLIC OR PRIVATE SECTOR, WRITE THE NAME OF THE PLACE.<br><br>________________<br>(NAME OF PLACE)	*PUBLIC SECTOR*<br>GOVERNMENT HOSPITAL . . . . . 11<br>GOVT. HEALTH CENTER . . . . . 12<br>GOVT. DISPENSARY . . . . . 13<br>OTHER PUBLIC SECTOR ________ 18<br>(SPECIFY)<br>*PRIVATE MEDICAL SECTOR*<br>PRIVATE HOSPITAL/CLINIC/ PRIVATE DOCTOR . . . . . 21<br>OTHER PRIVATE SECTOR ________ 28<br>(SPECIFY)<br>OTHER ________ 98<br>(SPECIFY)	
614	In which district is this place?	KASESE DISTRIC . . . . . 1<br>RUBIRIZI DISTRI . . . . . 2<br>KAMWENGE DISTRIC . . . . . 3<br>OTHER ________ 8<br>(SPECIFY)	

615	What is the name of this place?<br><br>SEE CODES OF FACILITIES IN ANNEX	______ (SPECIFY) [ ][ ]	→ END
616	Are you still followed up for the AIDS infection?	YES . . . . 1<br>NO 2	 → 621
617	When was your last consultation?<br><br>(IF NEEDED CHECK ON THE HEALTH BOOKLET)	MONTH . . . . [ ][ ]<br>DON'T KNOW . . . . 99<br>YEAR . . . . . . [ ][ ][ ][ ]<br>DON'T KNOW 9999	
618	Where are you now receiving care?<br><br>PROBE TO IDENTIFY THE TYPE OF SOURCE.<br><br>IF UNABLE TO DETERMINE IF PUBLIC OR PRIVATE SECTOR, WRITE THE NAME OF THE PLACE.<br><br>______<br>(NAME OF PLACE)	*PUBLIC SECTOR*<br>GOVERNMENT HOSPITAL . . . . 11<br>GOVT. HEALTH CENTER . . . . 12<br><br>OTHER PUBLIC SECTOR ______ 18<br>(SPECIFY)<br><br>*PRIVATE MEDICAL SECTOR*<br>PRIVATE HOSPITAL/CLINIC/ PRIVATE DOCTOR . . . . 21<br><br>OTHER PRIVATE MEDICAL SECTOR ______ 28<br>(SPECIFY)<br><br>OTHER ______ 98<br>(SPECIFY)	
619	In which district is this place?	KASESE DISTRIC . . . . 1<br>RUBIRIZI DISTRI . . . . 2<br>KAMWENGE DISTRIC . . . . 3<br>OTHER ______ 8<br>(SPECIFY)	
620	What was the name of this place?<br><br>SEE CODES OF FACILITIES IN ANNEX	______ (SPECIFY) [ ][ ]	→ END
621	Why did you stop?	NO ONE WAS ATTENDING 1<br>STOCK OUT 2<br>UNFRIENDLY STAFF 3<br>SPONTANEOUS 4<br>ADVISED TO STOP 5<br>THOUGHT I WAS CURED/ FEEL GOOD 6<br>SIDE EFFECT 7<br>MOVED AWAY 8<br>TRANSPORT COST 9<br>STOP PMTCT 10<br><br>OTHER ______ 98<br>(SPECIFY)	

INTERVIEWER'S OBSERVATIONS

TO BE FILLED IN AFTER COMPLETING INTERVIEW

COMMENTS ABOUT RESPONDENT:

COMMENTS ON SPECIFIC QUESTIONS:

ANY OTHER COMMENTS:

SUPERVISOR'S OBSERVATIONS

NAME OF SUPERVISOR: ______________________ DATE: ______________________

EDITOR'S OBSERVATIONS

NAME OF EDITOR: ______________________ DATE: ______________________
